# Leveraging Identity-by-Descent for Accurate Genotype Inference in Family Sequencing Data

**DOI:** 10.1371/journal.pgen.1005271

**Published:** 2015-06-04

**Authors:** Bingshan Li, Qiang Wei, Xiaowei Zhan, Xue Zhong, Wei Chen, Chun Li, Jonathan Haines

**Affiliations:** 1 Department of Molecular Physiology & Biophysics, Vanderbilt University, Nashville, Tennessee, United States of America; 2 Vanderbilt Genetics Institute, Vanderbilt University, Nashville, Tennessee, United States of America; 3 Center for Quantitative Sciences, Vanderbilt University, Nashville, Tennessee, United States of America; 4 Quantitative Biomedical Research Center, University of Texas Southwestern Medical Center, Dallas, Texas, United States of America; 5 Department of Pediatrics, University of Pittsburgh, Pittsburgh, Pennsylvania, United States of America; 6 Institute of Computational Biology, Department of Epidemiology and Biostatistics, Case Western Reserve University, Cleveland, Ohio, United States of America; University of Oxford, UNITED KINGDOM

## Abstract

Sequencing family DNA samples provides an attractive alternative to population based designs to identify rare variants associated with human disease due to the enrichment of causal variants in pedigrees. Previous studies showed that genotype calling accuracy can be improved by modeling family relatedness compared to standard calling algorithms. Current family-based variant calling methods use sequencing data on single variants and ignore the identity-by-descent (IBD) sharing along the genome. In this study we describe a new computational framework to accurately estimate the IBD sharing from the sequencing data, and to utilize the inferred IBD among family members to jointly call genotypes in pedigrees. Through simulations and application to real data, we showed that IBD can be reliably estimated across the genome, even at very low coverage (e.g. 2X), and genotype accuracy can be dramatically improved. Moreover, the improvement is more pronounced for variants with low frequencies, especially at low to intermediate coverage (e.g. 10X to 20X), making our approach effective in studying rare variants in cost-effective whole genome sequencing in pedigrees. We hope that our tool is useful to the research community for identifying rare variants for human disease through family-based sequencing.

## Author Summary

To identify disease variants that occur less frequently in population, sequencing families in which multiple individuals are affected is more powerful due to the enrichment of causal variants. An important step in such studies is to infer individual genotypes from sequencing data. Existing methods do not utilize full familial transmission information and therefore result in reduced accuracy of inferred genotypes. In this study we describe a new method that infers shared genetic materials among family members and then incorporate the shared genomic information in a novel algorithm that can accurately infer genotypes. Our method is particularly advantageous when inferring low frequency variants with fewer sequence data, making it effective in analyzing genome-wide sequence data. We implemented the algorithm in a computationally efficient tool to facilitate cost-effective sequencing in families for identifying disease genetic variants.

## Introduction

DNA sequencing is being routinely carried out to identify genetic factors, rare variants in particular, associated with human disease. It has been successful in identifying causal variants for Mendelian disease [1,2], and continues to be a powerful approach to uncovering the genetic basis of rare disease [3]. For complex traits, however, detecting rare variant associations is challenging due to reduced power of statistical tests when the allele frequency is low [4,5]. Although large-scale sequencing of unrelated individuals has identified associated rare variants for some complex traits, such as lipid traits [6], this approach often revealed greater challenges in finding causal genes for complex traits [7,8]. Family sequencing provides a promising alternative for identifying rare variant associations due to the enrichment of causal variants in pedigrees. Recent studies demonstrated the effectiveness of sequencing families and identified associated rare variants for a variety of traits, including schizophrenia [9], Alzheimer [10], and hypertriglyceridemia [11]. These lines of evidence show that family studies are emerging as a powerful approach in the sequencing era to localize genetic factors for human disease, and will play a key role as a complementary approach to the population based design to help understand the genetic basis of complex traits.

A critical step in genetic analysis of family sequence data is to infer genotypes of individuals in pedigrees. For next-generation sequencing data, this is challenging due to base call error, alignment artifacts, possible allele dropout during library preparation and sequencing, especially at low coverage, among others. Although variant calling algorithms developed for unrelated individuals can be applied to family sequencing data, the accuracy is compromised due to the ignorance of family relatedness. Family-aware calling algorithms, e.g. Polymutt [12] and FamSeq [13], have improved accuracy over the standard methods but assume the same pedigree correlation structure for all sites and therefore ignore the actual identity-by-descent (IBD) sharing. For example, in a nuclear family with two siblings and their parents, the IBD sharing between the two siblings can be 0, 1 or 2 at a particular genomic region, with the probabilities being 0.25, 0.5 and 0.25 respectively, *a priori*. Polymutt and FamSeq assume such *a priori* probabilities for all genomics regions and thus inefficiently model the data when the actual IBD can be inferred. As a concrete example, assuming we know that at a particular position the two siblings share 2 alleles IBD, then their genotypes are identical at this locus and can be inferred with improved accuracy by merging their sequencing data, essentially doubling the sequencing depth. In general, knowledge of IBD sharing helps confine genotypes to be compatible to the IBD patterns in pedigrees and a variant calling framework that models IBD is expected to deliver improved performance over existing methods. Such a framework makes it feasible to design studies with reduced coverage, since data from shared haplotypes in a pedigree are efficiently combined to make reliable genotype calls. This will be particularly beneficial for whole genome sequencing, which is still prohibitively expensive for large-scale sequencing studies. Linkage-disequilibrium (LD)-based methods such as Beagle4 [14], Thunder [15] and SHAPEIT [16] have been extensively used for inferring genotypes from low-depth sequencing utilizing extensive LD among variants. Due to much reduced LD (in terms of r^2^) among rare variants as well as between rare and common variants, however, LD-based methods are expected to have reduced accuracy for rare than for common variants.

In this study, we develop and implement a variant calling framework that infers the IBD sharing (through the inheritance vector, see Methods) among family members directly from sequencing data, and utilizes the IBD sharing to jointly infer individual genotypes. The new software, Polymutt2, provides a complementary tool to our prior work (Polymutt) with improved performance for small to moderate pedigrees. By directly modeling the sequencing data, the IBD can be reliably inferred, even for extremely low coverage (e.g. 2X or below), making it a robust tool for sequencing data. In addition to unphased genotypes, when parental data are available haplotypes can be directly constructed from the sequencing data based on the best inferred IBD sharing with little compromise of accuracy compared to unphased genotypes obtained by incorporating the uncertainties of IBD inference. Mendelian error is extremely rare for unphased genotypes, and is completely eliminated for haplotype calls. Through both simulations and real data, we show that Polymutt2 significantly outperforms other tools, including GATK, Beagle4 and Polymutt, for genotype calling on pedigree data, especially for rare variants in low coverage data.

## Methods

The input to Polymutt2 is a variant calling format (VCF) [17] file, which contains candidate variant sites and genotype likelihood (GL) values, defined as the probability of observing the reads given a specific underlying genotype (see [12,18] for details). For family members that were not sequenced, all GL values are set to 1 for all underlying genotypes. Standard approaches to variant calling are likelihood-based methods, both in unrelated individuals [19–21] and pedigrees [12,13]. All these methods calculate GL values and call an initial set of variant sites and individual genotypes. Our framework first infers IBD sharing in pedigrees along the genome based on the GL values, and then uses the inferred IBD sharing to assess variant quality, to refine individual genotypes, and to generate haplotypes along the genome. In the current implementation, we assume that all variants are bi-allelic and that the two alleles are known.

### Inference of the distribution of inheritance vectors

Suppose we have a pedigree with *f* founders and *n* non-founders, with sequencing data on *M* variants across a chromosome. Without loss of generality, we also arrange the pedigree such that the first *f* members in the pedigree are founders. Define a binary inheritance vector [22] at variant *j* as *I*_*j*_ *= (p*_*1*_,*m*_*1*_,*…*,*p*_*n*_,*m*_*n*_ *)* for the *n* non-founders in this pedigree. Each of the entries describes the transmission of the paternal (*p*_*i*_) or the maternal (*m*_*i*_) allele, with 0 (or 1) indicating the grand-paternal (or the grand-maternal) allele being transmitted. Therefore an inheritance vector completely determines which of the 2*f* founder alleles were inherited by each nonfounder. There are *N = 2* ^*n*^ possibility inheritance vectors and let *v*_k_, *k* = 1,..,*N*, denote individual vectors. Let **R** denote data of all members in a family across all *M* variants, **R**_*j*_ be all reads at variant *j*, and *R*_*ij*_ be the reads in family member *i* at variant *j*. Similarly, let **G**_*j*_ denote the vector of genotypes at variant *j* and its *i*th entry *G*_*ij*_ = (*A*_1_, *A*_2_) be the ordered genotype of the *i*th member in the pedigree, where *A*_1_ and *A*_2_ represent the paternally and maternally transmitted alleles respectively. Assuming that recombination events are independent between all chromosome intervals, i.e. no crossover interference, the likelihood can be framed as a Hidden Markov Model [23], similarly as that in the Lander-Green algorithm [22]. Specifically, the likelihood of reads across all *M* variants in the pedigree can be calculated as

$$P(R)=\sum_{I_{1}}\ldots \sum_{I_{M}}P(I_{1})\prod_{j=2}^{M}P(I_{j}|I_{j-1})\prod_{j=1}^{M}P(R_{j}|I_{j})$$

The initial probability, *P*(*I*_*1*_), is assumed uniform across all *N = 2* ^*n*^ inheritance vectors. The transition between adjacent inheritance vectors, *P*(*I*_*j*_|*I*_*j-1*_), is calculated according to the recombination rate between the *j*th and *j-1*th variant, which can be calculated using the HapMap Phase II [24] genetic map. For variants not in the HapMap genetic map linear extrapolation will be used to approximate the genetic distance. The emission probability, *P(* **R**_*j*_ *|I*_*j*_ *)*, which is the probability of reads in all family members at locus *j* given the inheritance vector *I*_*j*_, can be calculated as

$$P(R_{j}|I_{j})=\sum_{G_{j}}P(R_{j},G_{j}|I_{j})=\sum_{G_{j}}P(R_{j}|G_{j},I_{j})P(G_{j}|I_{j})=\sum_{G_{j}^{founders}}\prod_{i=1}^{f+n}P(R_{ij}|G_{ij},I_{j})\prod_{i=1}^{f}P(G_{ij})$$ (1)

Here we assume as in other methods that the sequencing reads depend only on the underlying genotype so that *P*(**R**_***j***_|**G**_***j***_) can be factorized into the product of individual genotype likelihoods. Since an inheritance vector specifies precisely how the alleles were transmitted from founders to non-founders, the genotypes of the entire pedigree are determined by the ordered founder genotypes when the inheritance vector is known. Therefore the emission probability involves the summation of only the ordered founder genotypes, whose prior probabilities *P*(*G*_*ij*_) can be either obtained from the external sources, e.g. the 1000 Genome Project [25], or estimated based on the pedigree data using for example Polymutt [12], assuming Hardy-Weinberg equilibrium.

Since the inheritance vectors usually cannot be determined unambiguously, the goal here is to infer the posterior distribution of the inheritance vectors at each variant using the sequencing data from all *M* variants; that is, we aim to calculate P(*I*_*j*_|**R**). This can be achieved efficiently using the forward-backward procedure in HMM [23]. Let *α*_*j*_(*k*) denote the forward variable at variant *j* for *v*_*k*_ and *β*_*j*_(*k*) be the corresponding backward variable [23]. Then the posterior probability of *v*_*k*_ at variant *j* is

$$P(I_{j}=v_{k}|R)=\frac{\alpha_{j}(k)\beta_{j}(k)}{\sum_{k=1}^{N}\alpha_{j}(k)\beta_{j}(k)}$$ (2)

From the marginal distribution, the inheritance vector with the maximum posterior probability at variant *j*, denoted as *I*_*j*_^*marg*^, can be used to represent the inferred inheritance vector for each variant; the IBD sharing can be directly derived from *I*_*j*_^*marg*^ for any pair of family members. However, since *I*_*j*_^*marg*^ only maximizes the likelihood marginally at variant *j*, we infer a global optimal path of inheritance vectors along the genome through the Viterbi algorithm [23]; we use *I*_*j*_^*best*^ to denote the optimal inheritance vector at variant *j*.

### Selection of variants to construct the genetic map

The Lander-Green algorithm requires that variants are independent, i.e. not in linkage disequilibrium (LD). For sequencing data variants are usually correlated. Since only a limited number of recombination events are expected in a pedigree, it is neither feasible nor necessary to use all data. We built a companion tool to automatically select a subset of independent variants by LD pruning, a similar approach used in Plink and others [26,27]. In addition, we filtered variants in genomic regions that are prone to alignment artifacts, including segmental duplications, simple repeats and low complexity regions, and 50bp up and downstream of known insertions and deletions; these data were downloaded from UCSC genome browser (http://www.genome.ucsc.edu) and the 1000 Genomes Project. The final set of selected variants is used to contrast a sparse genetic map with high-quality variants for the inference of inheritance vectors, and the genetic distances of these variants are linearly extrapolated based on the HapMap Phase II genetic map [24]. The overall strategy is to build a sparse scaffold of inheritance vectors along the genome using the selected set of variants and utilize the scaffold to boost the variant calling accuracy for all variants. We use the term “scaffold variants” to refer to the sparse set of variants in the map file used to construct the inheritance vectors.

### Variant calling—Refining variant sites

We evaluate for each variant the evidence supporting the alternative allele in the data by calculating the posterior probability of being polymorphic. Specifically for each variant we calculate two probabilities, *P*(poly|**R**_j_,**R**) and *P*(mono|**R**_j_,**R**), representing the likelihood of polymorphism and monomorphism respectively given the data at the *j*th site and scaffold variants. We assume that for poly the two alleles are *A*_*ref*_ and *A*_*alt*_ and for mono only *A*_*ref*_ is present in the data. The posterior probability of polymorphism given the data is calculated as

$$\begin{array}{l} P(\text{poly}|R_{j},R)=\sum_{I_{j}}P(\text{poly}|I_{j},R_{j},R)P(I_{j}|R_{j},R)=\sum_{I_{j}}P(\text{poly}|I_{j},R_{j})P(I_{j}|R_{j},R)\\ \;=\sum_{I_{j}}\frac{P(R_{j}|I_{j},\text{poly}|P(\text{poly}|I_{j})}{P(R_{j}|I_{j})}P(I_{j}|R_{j},R)\\ \;=\;\sum_{I_{j}}\frac{P(R_{j}|I_{j},\text{poly})P(\text{poly})}{P(R_{j}|I_{j},\text{poly})P(\text{poly})+P(R_{j}|I_{j},\text{mono})P(\text{mono})}P(I_{j}|R_{j},R) \end{array}$$

The term *P*(**R**_*j*_|*I*_*j*,_ poly) is calculated based on Eq (1), and *P*(**R**_*j*_|*I*_*j*,_ mono) is simply the product of genotype likelihoods of homozygous reference allele across all family members at variant *j*. The prior probability of polymorphism, *P*(poly), is calculated as in Polymutt [12] and *P*(mono) = 1-*P*(poly). Briefly, in a sample with *N* founders in the absence of natural selection, according to coalescent theory the prior probability that a site includes non-reference alleles is $\theta \sum_{i\;=\;1}^{2N}\frac{1}{i}$, where θ is the population scaled mutation rate per site and is set to 1/1000 in this study. When variant *j* is one of the scaffold variants, P(*I*_*j*_ | **R**_*j*_, **R**) = P(*I*_*j*_ | **R**), which was obtained in (2). Then the Phred-scaled variant quality is calculated as VQ = -10*log_10_(1-*P*(poly|**R**_*j*_,**R**)). By construction, only a sparse subset of variants is included in the map file, and the vast majority of variants are located in the intervals of scaffold variants. In an interval within which a crossover occurred, the inheritance vectors on the two sides of the recombination point are different. Assigning wrong inheritance vectors to variants will not only produce wrong IBD sharing among family members but also greatly reduce variant calling accuracy. However it is unknown *a priori* in which intervals crossovers occurred and where exactly the breakpoint is if a crossover occurred. To address this issue, Polymutt2 calculates for each variant in scaffold intervals the posterior probabilities using the inheritance vectors on the left and right boundary separately, and takes the maximum value, P_*max*_(poly| **R**_*j*_,**R**), as the posterior probability of polymorphism. The Phred-scaled variant quality is calculated as VQ = -10*log_10_(1-*P*_*max*_(poly|**R**_*j*_,**R**)). The inheritance vectors are accordingly assigned to each of the variants in scaffold intervals. This assumes that there is at most one crossover event in any interval, which is reasonable given the limited number of expected recombination events per generation. As a result, the crossovers can be precisely located in intervals in which crossovers occurred.

### Variant calling—Inferring genotypes

After quantifying the variant quality as described above, the most likely inheritance vectors are assigned to each of the variants. The posterior probability of the genotypes for individual *i* for variant *j* can be calculated as

$$\begin{array}{l} P(G_{ij}|R_{j},R)=\sum_{I_{j}}P(G_{ij}|I_{j},R_{j},R)P(I_{j}|R_{j},R)\\ \;=\sum_{I_{j}}P(G_{ij}|I_{j},R_{j})P(I_{j}|R_{j},R)=\sum_{I_{j}}\frac{P(G_{ij},R_{j}|I_{j})}{P(R_{j}|I_{j})}P(I_{j}|R_{j},R) \end{array}$$ (3)

For a specific genotype *G*_*ij*_ = *g*, the term *P*(*G*_*ij*_,**R**_*j*_|***I***_*j*_) can be calculated using Eq (1) by considering only the terms where *G*_*ij*_ = *g*. For variants in intervals of scaffold variants, *P*(*I*_*j*_|**R**_*j*_,**R**) was obtained in calculating the variant quality as described in the previous section. The genotype with the maximum posterior probability *P*_*max*_(*G*_*ij*_|**R**_*j*_,**R**) is assigned to the individual, and the corresponding genotype quality is calculated as GQ = -10log_10_(1-*P*_*max*_(*G*_*ij*_|**R**_*j*_,**R**)).

Since the calculation is repeated for all individuals in the pedigree, the computation can be intensive for larger pedigrees. One remedy is to use inheritance vectors with the largest posterior probabilities in the calculation. Specifically, the top inheritance vectors with cumulative probabilities greater than a cutoff, e.g. 0.99, can be used in (3). At the extreme, a single best inheritance vector, *I*_*j*_^*best*^ or *I*_*j*_^*marg*^, can used to minimize the computation. Given the high accuracy of the inheritance vector inference (see Results), the increase of speed greatly outweighs the negligible loss of accuracy.

### Variant calling—Inferring haplotypes

When parental data available, we generate haplotypes along a chromosome by reconstructing the optimal ordered genotypes jointly for all family members at each position assuming that the inheritance vector is known. We use *I*_*j*_^*best*^ as the optimal inheritance vector for variant *j* inferred using the Viterbi algorithm as we described before. The posterior probability of each configuration of ordered genotypes at variant *j* given the sequencing data and *I*_*j*_^*best*^ is calculated as

$$\begin{array}{l} P(G_{1j},G_{2j},\ldots ,G_{(f+n)j}|I_{j}^{best},R_{j})\\ \;=\;\frac{P(R_{j}|G_{1j},G_{2j},\ldots ,G_{(f+n)j},I_{j}^{best})P(G_{1j},G_{2j},\ldots ,G_{(f+n)j}|I_{j}^{best})}{P(R_{j}|I_{j}^{best})}\\ \;=\;\frac{\prod_{i=1}^{f+n}P(R_{ij}|G_{ij})\prod_{G_{j}^{founder}}P(G_{j}^{founder})}{P(R_{j}|I_{j}^{best})} \end{array}$$

Here *P*(*R*_*ij*_|*G*_*ij*_) is the genotype likelihood calculated before, and the term $P(G_{1j},G_{2j},\ldots ,G_{(f+n)j}|I_{j}^{best})$ is simplified to $\prod_{G_{j}^{founder}}P(G_{j}^{founder})$ since given an inheritance vector the probability depends only on the ordered genotypes of founders. The terms $P(G_{j}^{founder})$ and $P(R_{j}|I_{j}^{best})$ were calculated in Eq (1). The goal is to obtain the posterior probability of each configuration of founder ordered genotypes, and assign the configuration with the maximum posterior to founders as well as nonfounders according to *I*_*j*_^*best*^. This is repeated for all positions and the haplotypes are automatically constructed by stitching the paternal and maternal alleles at each position along a chromosome. By construction, Mendelian error in haplotype calling is completely eliminated due to the Mendelian transmission dictated by the inheritance vector.

Note that the construction of haplotypes is based on the transmission of alleles from parents to offspring. When parental data are not available, such as in sibships, it is not possible to deduce the parental origin of the alleles and therefore haplotypes cannot be reconstructed.

### Simulations

We utilized the 1000 Genome Project [25] data to effectively capture the sequencing and mapping error. We generated the founders’ genomes by randomly selecting the CEU phased genotypes (March 2012 Phase 1 release). For non-founders, we simulated cross-overs in the parental haplotypes based on the genetic map in the Phase II HapMap data, and then generated offspring genotypes by randomly selecting one haplotype from each parent. To simulate realistic reads, we first generated paired-end 100bp fragments according to Poisson distribution on the genome, with the insert size following a Gaussian distribution with a mean of 400bp and a standard deviation of 50bp, and then simulated reads based on these fragments assuming a sequencing error rate of 0.01 per base. We used BWA [28] to align simulated reads to the reference of hg19 and carried out standard procedures for variant calling, including Indel-realignment and base quality recalibration using GATK and duplication removal using Picard (http://picard.sourceforge.net). The list of known Indels from the 1000 Genomes Project was provided to GATK for Indel re-alignment. We used GATK UnifiedGenotyper to infer variants and genotypes from sequencing. We then applied Polymutt, Polymutt2 and Beagle4 on the GATK-generated VCF files to refine the genotypes utilizing the GL values calculated by GATK and stored in the VCF file.

Pedigrees we investigated in this study include sibships of size 2 (Sib2), 4 (Sib4) and 6 (Sib6), nuclear families with 4 (Nuc4) and 6 (Nuc6) members, and an extended pedigree with 10 individuals, which is the same as the pedigree investigated in Polymutt [12]. For each pedigree structure, we simulated 20 families at coverage ranging from 2X to 30X. For the Nuc6 we simulated additional 50 and 100 pedigrees to investigate the trend of the genotype calling accuracy of rare variants for increasing numbers of sequenced families. Genotype calling was performed using GATK, Polymutt, Polymutt2 and Beagle4 for each simulated dataset. Note that for trios Polymutt2 and Polymutt are equivalent, and therefore we omitted the investigation of trios in this study.

### Performance evaluation metrics

We used two metrics to measure the accuracy of genotype calling. The first is the false negative rate (FNR), defined as the percentage of true genotypes that are called into incorrect genotypes; this measures the sensitivity of the calling and is equal to 1-sensitivity. The second metric is the false discovery rate (FDR), defined as the percentage of called genotypes that are different from the true genotypes; this measures the specificity of the calling; this measures the specificity of the calling and corresponds to 1-precision. A good algorithm is expected to have low values of both FNR and FDR. We used GQ to filter low quality genotype calls and specifically we used GQ = 3 for Polymutt2 and Polymutt, GQ = 5 for Beagle4 and GQ = 10 for GATK; due to different calculations of GQ in these algorithms we found that these filtering criteria have reasonable FNR and FDR values. These criteria were used for all simulated data. Note that when no filtering is used FNR and FDR are the same for overall genotypes, and the difference is due to differential filtering based on GQ cutoffs. For heterozygotes, which are of particular interest in studying rare variants, however, both FNR and FDR are critical metrics to evaluate, as FNR can be made artificially low by aggressive calling of heterozygotes, which will results in high FDR, and conversely conservative calling of heterozygotes can lead to low FDR and high FNR.

## Results

### Accuracy of inferred inheritance vectors

**Fig 1:**
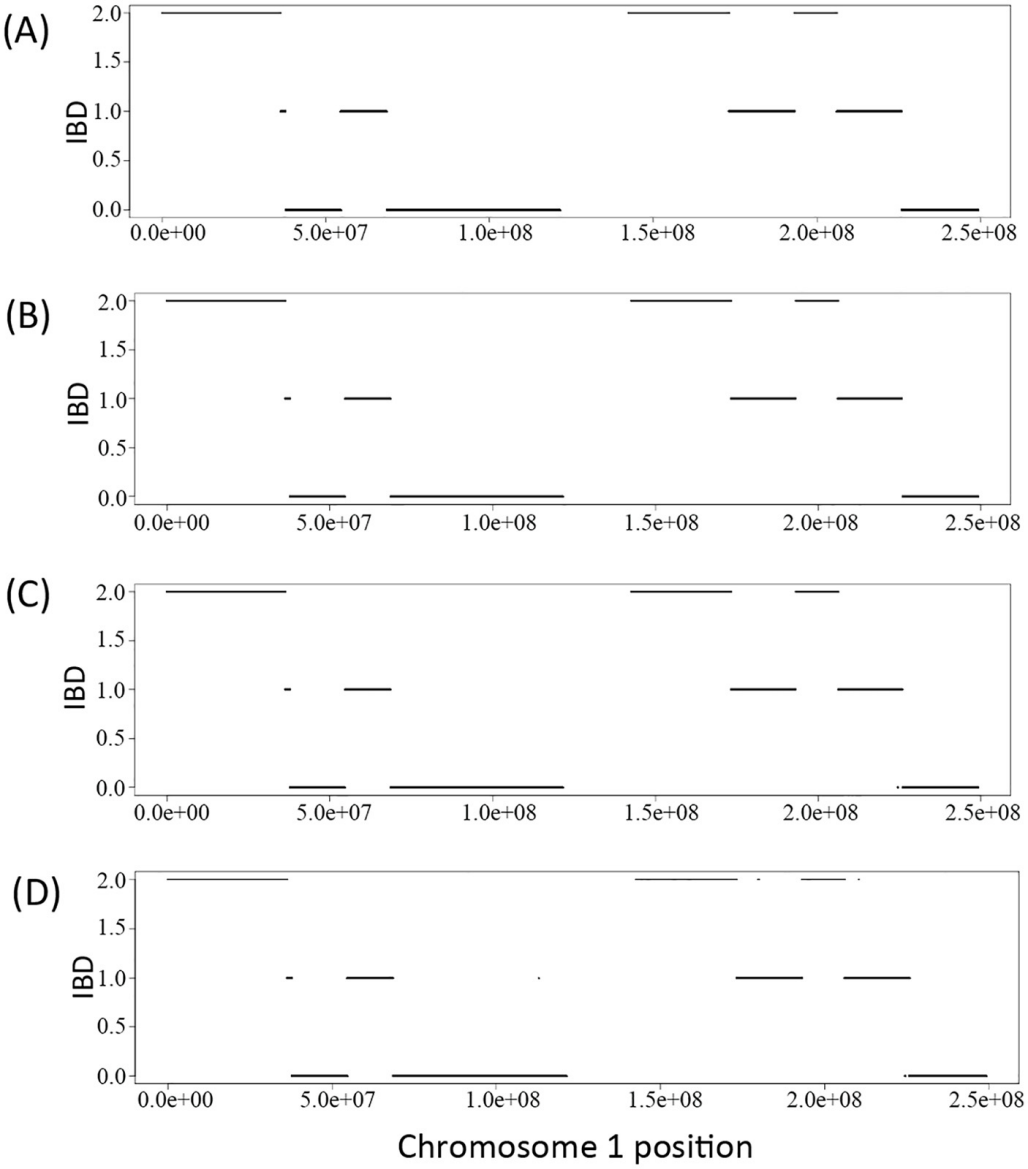
The IBD of the two siblings in a Nuc4 pedigree along the chromosome 1. A) is the simulated true IBD of the siblings, B), C) and D) are the inferred IBD at 30X, 15X and 2X respectively.

We derived the IBD sharing at each position between a pair of family members in a pedigree based on the inferred inheritance vector obtained via the Viterbi algorithm. Fig 1A) shows the simulated true IBD sharing of the two siblings in the Nuc4 pedigree along chromosome 1 and Fig 1B and 1C and 1D) show the inferred IBD sharing of the same two siblings at coverage of 30X, 15X and 2X, respectively. From the comparison, we can see that the inferred IBD is extremely close to the true IBD at various coverage, indicating the high accuracy of the inference of inheritance vectors based on sequencing data. Interestingly, for coverage as low as 2X, the accuracy of inferred IBD is not jeopardized (Fig 1D). The high accuracy of inheritance vector inference warrants the increased accuracy of genotype calling when the IBD sharing is utilized to infer genotypes.

### Genotype calling accuracy—Overall

**Fig 2:**
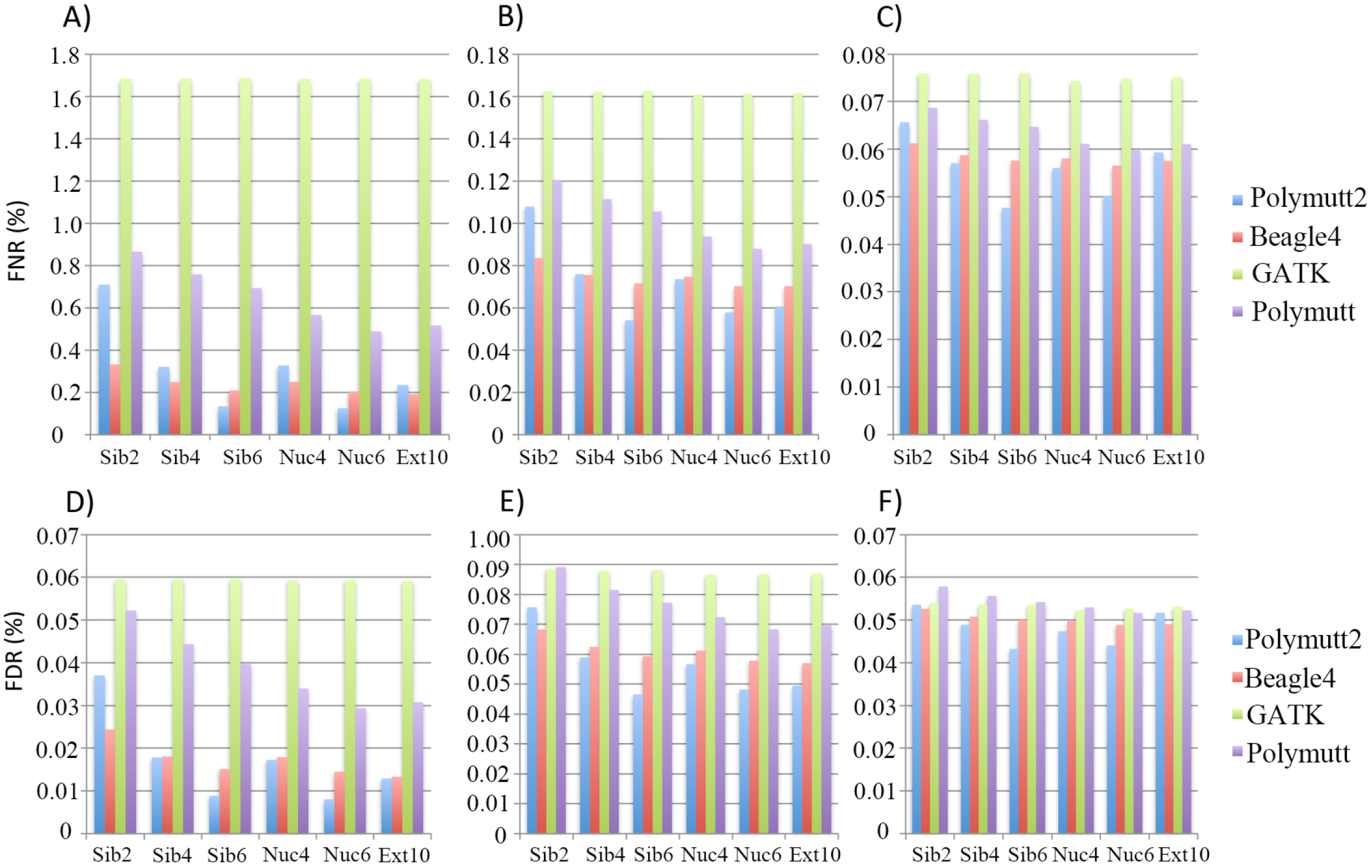
The FNR (%) (panel A, B and C) and FDR (%) (panel D, E and F) of the overall genotypes in 6 pedigrees from four callers (Polymutt2, Beagle4, GATK and Polymutt). Panels A, B and C show FNR (%) for sequencing coverage of 10X, 20X and 30X, and panels D, E and F show the FDR (%) for the same set of coverage.

Fig 2 shows FNR and FDR values for four calling algorithms (GATK, Polymutt, Polymutt2 and Beagle4) on overall genotypes for various pedigrees and sequencing coverage. Polymutt2 significantly outperforms Polymutt and GATK, in terms of both FNR and FDR, and the advantages are more pronounced when pedigree members are more related or coverage is low (Fig 2). For example, at 10X, the FNR values for Polymutt2 for sibships of size 2, 4 and 6 are 0.71%, 0.32% and 0.17%, respectively, while the FNR values for GATK are similar across all pedigree types with a mean value of 1.65% (Fig 2A). The FDR follows the same patterns (Fig 2D). On the other hand, the relative performance of Polymutt2 vs. Beagle4 depends on pedigree types, and for pedigrees with limited IBD sharing Beagle4 outperformed Polymutt2. For example for Sib2 Beagle4 calls have smaller FNR and FDR for all sequencing coverage investigated (Fig 2). For pedigrees with increased IBD sharing, Polymutt2 has either comparable (e.g. for Sib4, Nuc4 and Ext10) or better (e.g. for Nuc6 and Sib6) genotype calling accuracy, and the advantage of Polymutt2 over Beagle4 becomes more manifest with increasing IBD sharing in pedigrees such as Sib6 and Nuc6 (Fig 2). If we compare callers without Polymutt2, Beagle4 consistently outperformed GATK and Polymutt in terms of both FNR and FDR for all pedigrees and sequencing coverage (Fig 2). It is worth noting that although Beagle4 does not explicitly model family inheritance the algorithm is able to leverage the IBD sharing implicitly so that the genotype accuracy is improved for pedigrees with more IBD sharing. For example, the error rates of Beagle4 calls in Nuc6 are lower than those in Nuc4 calls (Fig 2). For all algorithms it is clear that sequencing coverage is the key factor influencing the calling accuracy (Fig 2), and for coverage of 30X the genotype calls are accurate to an extent that the differences among all callers become noncritical (Fig 2C and 2F). In the following sections we only presented results on 10X and 20X simulated data representing intermediate sequencing coverage to investigate the gain of explicit modeling of IBD sharing for genotype calling in such settings.

### Genotype calling accuracy—Heterozygotes

We next investigated the accuracy of the heterozygous genotypes, which are of particular interest for rare variants. S1 Fig shows the error rates for various pedigrees at different coverage. Consistent with the accuracy of overall genotypes (Fig 2), Polymutt2 and Beagle4 dramatically reduce error rates across all pedigrees and coverage, compared to both GATK and Polymutt, and the reduction is more dramatic when more related individuals are sequenced (S1 Fig). For example, the FNR at 10X is 1% and 0.8% for Polymutt2 and Beagle4 respectively, and is increased to 1.8% for Polymutt and 4.4% for GATK (S1A Fig). The same magnitudes were observed for FDR at 10X (S1D Fig). Consistent with the overall genotypes, both Polymutt2 and Beagle4 achieved better accuracy for pedigrees with more IBD sharing (S1 Fig). Polymutt2 outperformed Beagle4 on pedigrees of Sib6 and Nuc6 due to explicit modeling of the extensive IBD sharing in such pedigrees (S1 Fig).

### Genotype calling accuracy—Heterozygotes of rare variants by allele frequency

**Fig 3:**
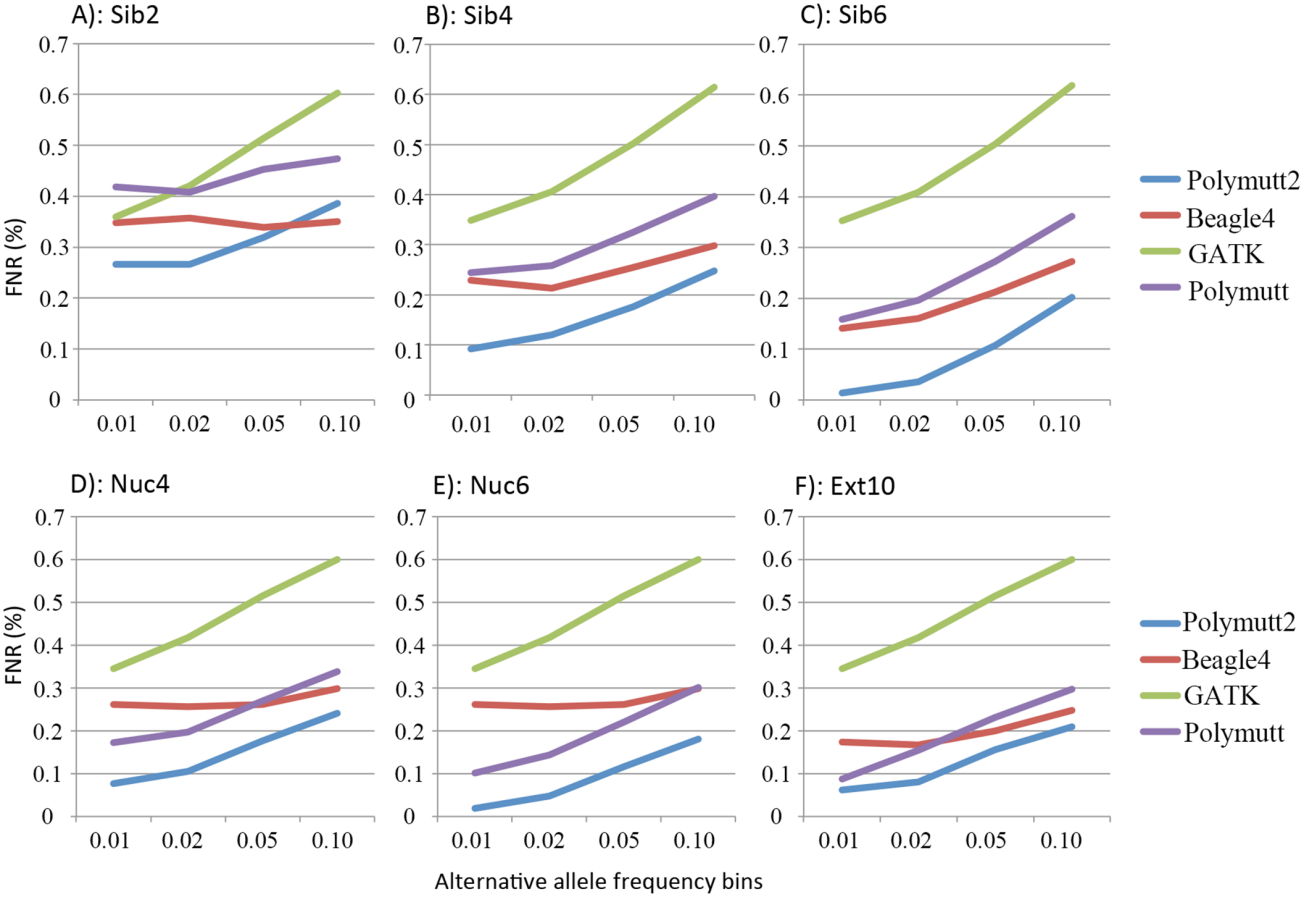
The FNR (%) of the heterozygotes in 6 pedigrees from four callers (Polymutt2, Beagle4, GATK and Polymutt) for variants with alternative allele frequencies in 4 bins in the range of [0, 0.1] at sequencing coverage of 20X. Results of different pedigrees are shown in panel A) for Sib2, B) for Sib4, C) for Sib6, D) for Nuc4, E) for Nuc6 and F) for Ext10.

The major interest of sequencing studies, especially in family designs, is to identify rare variants associated with disease. Accurate heterozygote calling is of particular importance due to the challenges associated with rare variant inference from sequencing. We specifically investigated the heterozygote accuracy across different bins of alternative allele frequencies, in the range of (0,0.01], (0.01, 0.02], (0.02, 0.05] and (0.05, 0.1]. Fig 3 shows the FNR of heterozygotes for sequencing coverage of 20X and S2 Fig shows the corresponding FDR measurements. It is clear that Polymutt2 achieved superior accuracy compared to others, and for all pedigrees except Sib2 Polymutt2 has lowest error rates in terms of both FNR and FDR across all bins for variants with frequency below 0.1 (Fig 3 and S2 Fig). For Sib2, which is the simplest pedigree with limited IBD sharing, although Beagle4 achieved better accuracy on overall genotypes and heterozygotes (Fig 2 and S1 Fig), Polymutt2 outperformed Beagle4 for variants with frequencies below 0.05 (Fig 3A and S2 Fig). Consistent with overall genotypes, the advantage of Polymutt2 increases for pedigrees with more IBD sharing (Fig 3 and S2 Fig).

**Fig 4:**
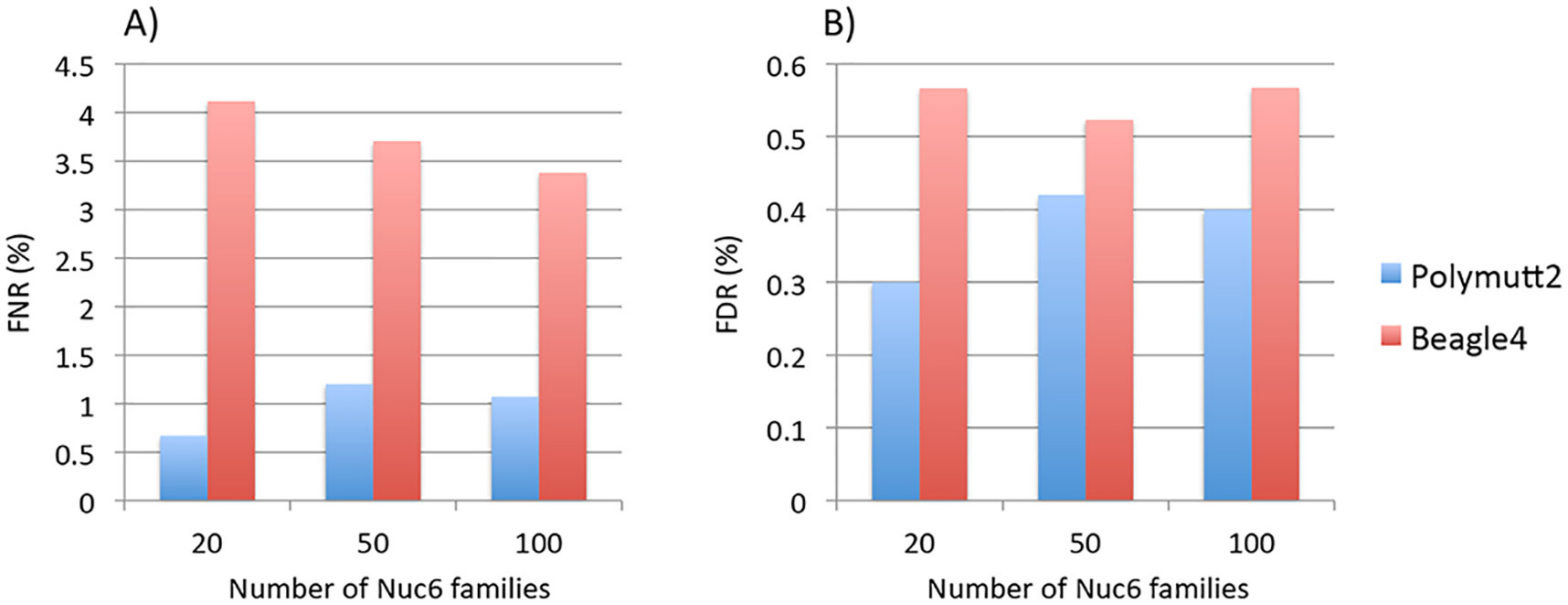
The FNR (%) in Panel A) and FDR (%) in Panel B) of heterozygous genotypes at variant sites with MAF<0.02 for Polymutt2 and Beagle4 calls for different numbers of simulated Nuc6 pedigrees at 10X coverage.

To investigate the effect of increasing numbers of sequenced families on rare variant calling, we simulated additional 50 and 100 Nuc6 families at 10X coverage and carried out genotype calling for both Polymutt2 and Beagle4. It is evident that the accuracy of Beagle4 heterozygous calls improves with increasing numbers of families for variants with MAF<0.02 (Fig 4). The improvements, however, do not seem to be dramatic, probably due to the limited LD among rare variants even for data with 100 families. In comparison, Polymutt2 achieved superior accuracy than Beagle4 for heterozygotes with MAF<0.02, for both FNR and FDR (Fig 4), indicating the advantages of Polymutt2 over Beagle4 for calling rare variants.

### Genotype calling accuracy—Phased genotypes

The genotype accuracy of phased genotypes (haplotypes) is similar to the unphased genotypes, although on average the error rates are slightly higher for phased genotypes. For example, for Nuc4 pedigrees at 15X, the FNR of overall genotypes is 0.30% for phased genotypes and is 0.28% for unphased genotypes; the corresponding FDR is 0.09% and 0.10% respectively. For heterozygotes, the FNR is 0.53% and 0.51% for phased and unphased genotypes respectively, with corresponding FDR values being 0.10% and 0.08%.

### Mendelian inconsistency

**Fig 5:**
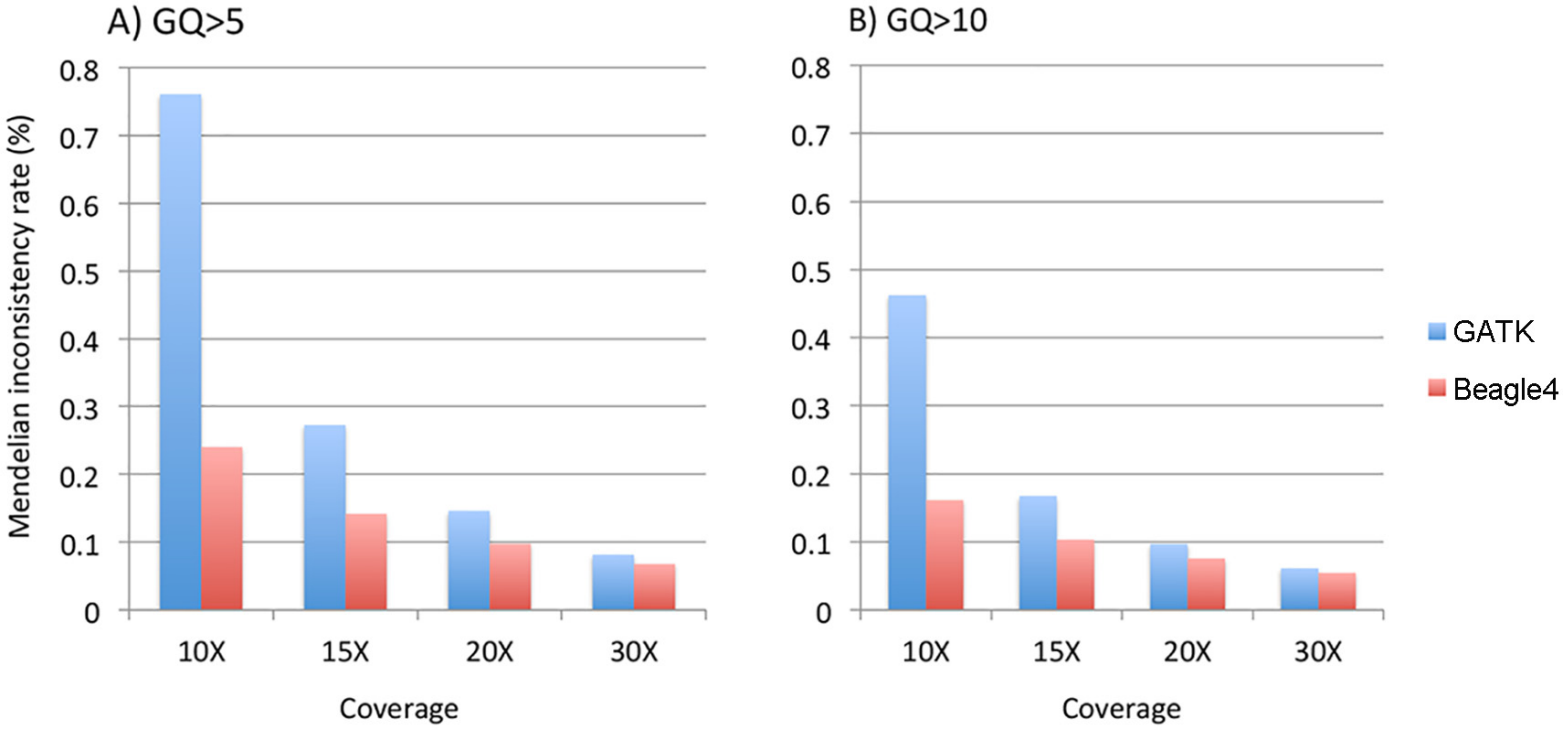
The average Mendelian inconsistency rates of Beagle4 and GATK calls per parents-offspring trio in the Nuc4 pedigrees at sequencing coverage of 10X, 15X, 20X when GQ = 5 (panel A) or GQ = 10 (panel B) was used to filter low quality genotypes.

We calculated the Mendelian inconsistency (MI) rate as the percentage of parent-offspring trios in which the genotypes violate the Mendelian transmission law. Pedigrees were divided into individual trios for the calculation. We used the minimum GQ of the genotypes in a trio as the filtering criteria to calculate MI rates on relatively high quality genotype calls. When either GQ 5 or 10 was used, both GATK and Beagle4 calls showed considerable Mendelian inconsistencies across various sequencing coverage (Fig 5). For example, at minimum GQ of 5, the MI rate is 0.76% for GATK at 10X, and 0.15% when coverage was increased to 20X. Although Beagle4 achieved reduced MI rates than GATK, there are still noticeable Mendelian inconsistencies in Beagle4 calls (Fig 5). When the minimum GQ of 10 was used there are still appreciable Mendelian inconsistencies in both GATK and Beagle4 calls (Fig 5). On the other hand, the MI rates for Polymutt were extremely low, e.g. <10^–6^ for all scenarios shown above, consistent with the previous report [12]. Strikingly, no Mendelian inconsistencies were observed in Polymutt2 calls in the same settings.

### Application to real data

We downloaded the whole genome sequencing data in CEPH pedigree 1463 generated on the Illumina HiSeq platform (http://www.illumina.com/platinumgenomes/). We selected a Nuc6 sub-pedigree for the analysis, which consists of four siblings (NA12879, NA12880, NA12881 and NA12882) and their parents (NA12877 and NA12878). The sequencing coverage of these samples is ~50X. We followed the best-practice procedure for variant calling as we did for simulated data. Since there is only a single family with a few individuals, we used the 1000 Genomes Project reference panel when running Beagle4 (downloaded from Beagle4 website) on this pedigree to leverage the extensive LD in the panel. To have a fair comparison of Polymutt2 with Beagle4, we ran Polymutt2 using the allele frequencies derived from the same reference panel. Based on simulation results it is clear that at high coverage over 30X the accuracy measures of all callers are satisfactory. Our major goal here is to investigate to what extent genotype calls from various callers with a subset of data can recover the original high depth sequencing data. We first created a gold-standard callset from the original high-depth data by taking the consensus of genotype calls from GATK, Polymutt, Polymutt2 and Beagle4; this call set contains genotypes that are agreed by all 4 callers. GATK and Polymutt infer allele frequencies from the sequence data only, and due to the small sample size of the pedigree the estimates are not reliable. Here we focused only on the comparison of Polymutt2 and Beagle4, two competing methods based on simulated data. Specifically, we randomly extracted 30% and 15% of the reads from the original alignment, corresponding to ~15X and ~7.5X of coverage, and carried out variant calling using both Polymutt2 and Beagle4. For each of the two callers we calculated FNR and FDR using the gold-standard callset. We also compared their performance stratified by allele frequencies, which were calculated based on the same reference panel used in Beagle4. Since genotype filtering has a strong impact on FNR and FDR, e.g. aggressive filtering results in low FDR and high FNR and vice versa, we calculated the two measurements using GQ values from 3 to 30 and plotted them in FNR-FDR curves to represent genotype accuracy with a wide range of filtering. This is an objective way of comparing genotype accuracy and a curve completely underneath the other indicates consistent high accuracy of genotype calls for all GQ cutoffs in the range of 3 to 30.

**Fig 6:**
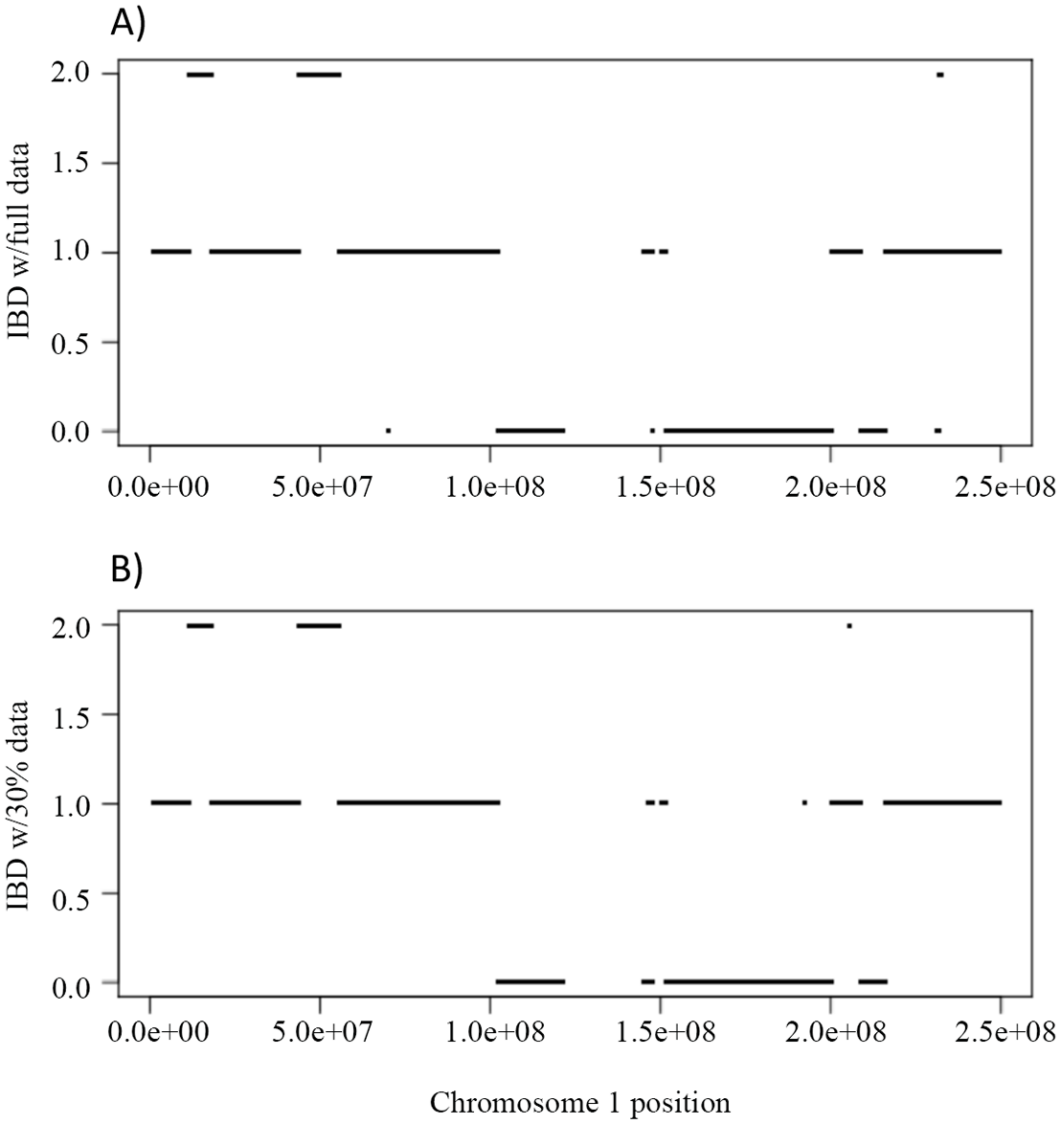
The IBD sharing and genotype accuracy of Illumina HiSeq sequencing data on chromosome 1 in a Nuc6 subpedigree from the CEPH 1463. A) shows the IBD of the siblings NA12879, NA12880 when the full data (~50X) were used to infer the inheritance vectors. B) shows the corresponding IBD when 30% (~15X) data were used.

First we evaluated the inference of IBD using the full and partial data. Fig 6A shows the IBD of NA12879 and NA12889 across chromosome 1 using full data, and Fig 6B shows the corresponding IBD when 30% data were used. The IBD sharing is very similar using full and partial data with only a few discrepancies (Fig 6A and 6B), indicating the robustness of the inheritance vector inference.

**Fig 7:**
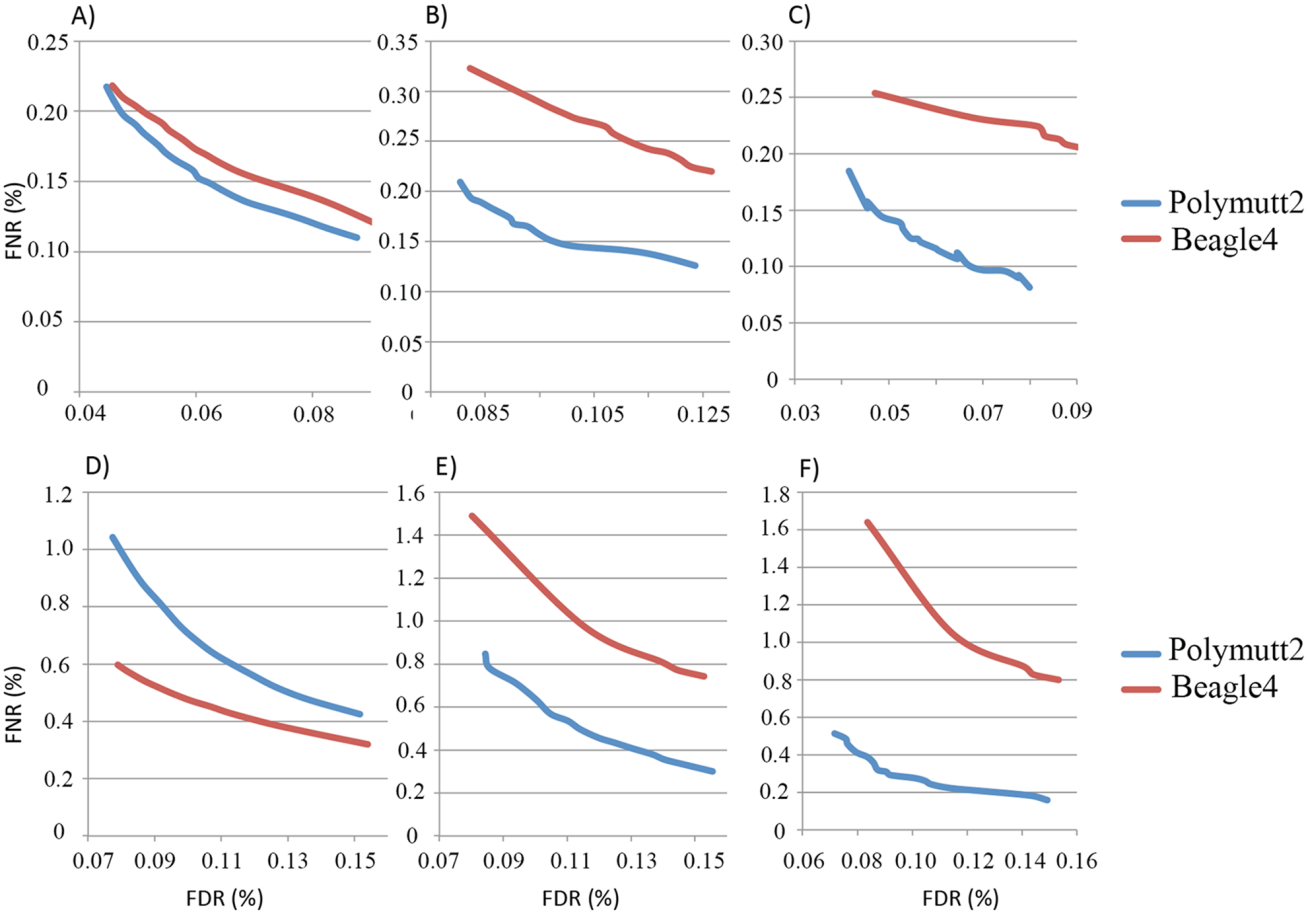
FNR (%) vs. FDR (%) curves of the overall genotypes of Polymutt2 and Beagle4 calls when 30% (~15X, panel A, B and C) or when 15% (~7.5X, panel D, E and F) of the original data were used for genotype calling. Panel A) and D) are for all variants, and panel B) and E) are for variants with MAF<0.01 and panel C) and F) are for variants with MAF<0.05. See *Application to real data* for the details on the pedigree and the calculation of error rates.

For overall genotypes with 30% of the data (~15X), Polymutt2 calls achieve greater concordance with the gold standard callset than Beagle4, as manifested by the reduced error rates in the FNR-FDR curves (Fig 7A). When we focused on variants with low frequencies, the advantage of Polymutt2 over Beagle4 is more pronounced (Fig 7B and 7C). For example, with allele frequency <0.1, the FNR-FDR curve of Polymutt2 is more separated from that of Beagle4, and with allele frequency <0.05 we observe further decreasing error rates in Polymutt2 calls than in Beagle4 calls. Interestingly, when 15% of data (~7.5X) were used, Beagle4 calls have better overall accuracy than Polymutt2 (Fig 7D), probably due to the increased contribution of LD relative to sequencing data on the genotype calls. However, when we focused on low frequencies variants with allele frequency <0.1 and <0.05, Polymutt2 still greatly outperformed Beagle4 (Fig 7E and 7F).

When we focused on heterozygotes, Polymutt2 and Beagle4 calls have similar accuracy when all variants were considered with both 30% and 15% of the data (S3A and S3D Fig). When the analyses were carried out on variants with allele frequency <0.1 and <0.05, it is clear that Polymutt2 generated more accurate heterozygous calls than Beagle4 (S3 Fig).

We observed considerable MI rates in Beagle4 calls with both ~15X and ~7.5X data. For example, at ~15X the MI rates are 0.14% and 0.09% when the minimum GQ was set to 5 and 10, respectively. The corresponding MI rates at ~7.5X are 0.15% and 0.1%. When we focused on low frequency variants, the MI rates in Beagle4 calls are noticeably increased. For example, for variants with allele frequency <0.1 at ~15X, the MI rates are 0.27% and 0.21% for GQ cutoffs of 5 and 10, respectively, indicating that increased genotype error rates associated with low allele frequencies in Beagle4 calls resulted in higher MI rates. On the other hand, as a direct comparison, we did not observe Mendelian error in Polymutt2 calls in all of these scenarios investigated, indicating the extremely low Mendelian error rate in Polymutt2 calling in real data.

## Discussion

Sequencing pedigrees has shown its effectiveness in identifying rare variants associated with human disease, and is expected to continue in gene mapping for complex traits in complement to population-based designs. In addition, family designs are not prone to population stratification, which may be more challenging to control for rare variants [29]. In this study we developed a new tool, Polymutt2, for accurate inference of inheritance vectors and genotype calling for pedigree sequencing data. Through both simulations and application to real data, the new tool achieves markedly improvement of genotype calling accuracy compared to the standard method (GATK) and a family-aware algorithm (Polymutt), as well as an LD-based caller (Beagle4), especially for low frequency variants. The advantages are mainly due to the explicit modeling of the IBD among family members and then the incorporation of the IBD information in genotype calling. This framework efficiently utilizes the relatedness by combining sequencing data from shared haplotypes among all family members across the genome. For the inference of inheritance vectors, which is critical for genotype and haplotype calling, we directly model the sequencing data in an effort to increase the robustness via the incorporation of sequencing error and depth of coverage in the likelihood calculation. Additional increase in performance comes from the careful selection of the scaffold variants in modeling the inheritance vectors. We plan to refine the selection of scaffold variants to further minimize the inadvertent effect of alignment artifacts on the inference of the inheritance vector, e.g. by exploring the alignment files to filter sites with nearby Indels, homopolymers, allelic imbalances, strand and cycle bias, among others.

Compared to Polymutt and GATK, Polymutt2 has increased accuracy of genotype calling from all aspects. This is rather unsurprising given that Polymutt2 uses extra information than the other two callers. On the other hand, Polymutt2 and Beagle4 use orthogonal information, i.e. the explicit modeling of IBD sharing in Polymutt2 vs. the utilization of LD among variants in Beagle4 for variant calling. Since the LD (r^2^ in this context) between rare variants and between rare and common variants is low, the effectiveness of LD-based calling for rare variants is reduced. Although for pedigrees with limited IBD sharing (e.g. sibpairs) Beagle4 outperformed Polymutt2 when considering all genotypes, Polymutt2 still achieved increased accuracy in calling rare variants. In addition, Mendelian inconsistency in LD-based calls, especially for rare variants, which are usually analyzed in groups, may have inadvertently impact on association analysis since the effect of Mendelian error in individual variants may be aggregated and amplified. As the major focus in sequencing is to identify rare variants we hope that Polymutt2 is useful for gene mapping of rare variants for complex disease.

Although most current studies focus on exome sequencing, multiple lines of evidence indicate the need for whole genome sequencing to identify risk factors for complex disease. Given the current cost, it is still not practical to carry out large-scale high coverage whole genome sequencing studies. Our tool makes it feasible for whole genome sequencing of pedigrees with reduced coverage. On the other hand, Polymutt2 is equally effective in targeted sequencing of small genomic regions, such as peaks revealed in linkage analysis, since the inheritance vectors are expected to be reliably inferred by modeling the shallow off-target sequences across the genome.

Since the haplotype calling in Polymutt2 is based on inheritance vectors only, the phase cannot be determined for some variants in which parents and offspring are heterozygotes. In such case, the phases are randomly assigned and should not be used without further information. Although LD can be used to phase such variants in trios [30], the simultaneous modeling of LD and inheritance vectors in complex pedigrees is computationally challenging. On the other hand, this limitation in Polymutt2 has little impact on the analysis since for rare variants, which are the major focus of sequencing studies, such situations are extremely uncommon. Note that for such variants only phasing is affected but the accuracy for both phased and unphased genotypes benefits equally from IBD modeling as other variants.

Since the Lander-Green algorithm is the major component for the inference of the inheritance vectors, the computation is linear with respect to the number of variants but can be explosive when pedigrees get large. For a pedigree with *f* founders and *n* nonfounders, the possible number of inheritance vectors is 2^2n^. Due to the lack of phasing information of founder alleles, these inheritance vectors are organized into 2^*f*^ equivalent classes so that only 2^2n-f^ inheritance vectors are required to model, a factor of 2^*f*^ reduction in terms of computation and storage [31]. Furthermore, we implemented the Fast Fourier Transformation in the Lander-Green algorithm [32], which reduces the computation from *O*(*N* ^2^) to *O*(*N*log*N*)in the HMM, where *N* is the number of inheritance vectors. Even with these speedup techniques, however, the computation can be still very challenging. To further mitigate the problem, we implemented the software using multi-threads so that the computation can be parallelized when possible. The current implementation can handle simple pedigrees efficiently. For example, for sibpairs and sibships of size 4, and nuclear families of size 4 and 6, the average time per family using 8 threads for chromosome 1 whole genome sequencing is on the scale of minutes. For sibships of size 6 the time is significant increased and it took over an hour to finish variant calling per family. For pedigree of Ext10 the time is even further increased to over 10 hours to get marginal calls. If computing is limited an option is to use only inheritance vectors with highest posterior probabilities for such pedigrees; for example using the single best inheritance vector the computing is a few minutes. For pedigrees beyond the exact calculation of the likelihoods, Monte Carlo approaches [33–35] are necessary, which is beyond the scope of the current study and will be explored in the future.

In the inference of inheritance vectors, we selected the scaffold variants by LD pruning. The results reported in the article were based on the maximum correlation coefficient of R^2^ = 0.2. We also investigated other thresholds to evaluate the sensitivity of the results to the LD pruning. Specifically we used cutoffs of 0.1 and 0.5 and observed similar results as 0.2, with the difference below 0.01% for most of pedigrees and coverage investigated in Figs 1 and 2, indicating the robustness of the framework to LD. This robustness makes it flexible to select scaffold makers without comprising the genotype calling accuracy.

With the comprehensive catalog generated by the 1000 Genomes Project, identifying known variants in study sample is generally very accurate. However calling novel variants for pedigrees is usually of particular interest. This remains challenging due to potential alignment artifacts. Unannotated structural variants are a major source of alignment artifacts, and when such artifacts do not follow Mendelian transmission laws the variant quality is expected to be dramatically reduced for such sites when the IBD sharing is imposed in the calculation of the likelihood. We believe that Polymutt2 is effective in filtering false novel variant candidates given its efficient use of allele sharing.

In our framework the increased accuracy of variant calling is due to the efficient use of the Mendelian inheritance. *De novo* mutations, however, violate the rule and make the inference of inheritance vector inaccurate. Although it is unlikely to include *de novo* mutations in scaffold variants, accidental inclusion of such variants makes the results not reliable. To avoid this situation, Polymutt2 internally checks the likelihood of *de novo* mutations during the calculation and if a strong violation of Mendelian inheritance is detected the algorithm ignores these variants so that the inheritance vectors can be robustly inferred. The current version of Polymutt2 is not designed to call *de novo* mutations and other methods (e.g. Polymutt and DeNovoGear [36]) should be used for that purpose.

Our tools were implemented in C++. The source code and company resources can be downloaded from the authors’ website (https://medschool.vanderbilt.edu/cgg). We hope that our user-friendly software packages are useful to the research community for pedigree sequencing studies to facilitate the identification of rare variants for human disease.

## Supporting Information

S1 Fig The FNR (%) (panel A, B) and FDR (%) (panel C, D) of the heterozygotes in 6 pedigrees from four callers (Polymutt2, Beagle4, GATK and Polymutt). Panels A and B show FNR (%) for sequencing coverage of 10X and 20X, and panels C and D show the FDR (%) for the same set of coverage.
(TIF)

S2 Fig The FDR (%) of the heterozygotes in 6 pedigrees from four callers (Polymutt2, Beagle4, GATK and Polymutt) for variants with alternative allele frequencies in 4 bins in the range of [0, 0.1] at sequencing coverage of 20X. Results of different pedigrees are shown in panel A) for Sib2, B) for Sib4, C) for Sib6, D) for Nuc4, E) for Nuc6 and F) for Ext10.
(TIF)

S3 Fig FNR (%) vs. FDR (%) curves of the heterozygotes of Polymutt2 and Beagle4 calls when 30% (~15X, panel A, B and C) or when 15% (~7.5X, panel D, E and F) of the original data were used for genotype calling. Panel A) and D) are for all variants, and panel B) and E) are for variants with MAF<0.01 and panel C) and F) are for variants with MAF<0.05. See *Application to real data* for the details on the pedigree and the calculation of error rates.
(TIF)
