# Supplementary figures and images for: Leveraging Identity-by-Descent for Accurate Genotype Inference in Family Sequencing Data
Source: PLoS Genet. 2015 Jun 4;11(6):e1005271. doi: 10.1371/journal.pgen.1005271 (PMC4456389; doi:10.1371/journal.pgen.1005271)

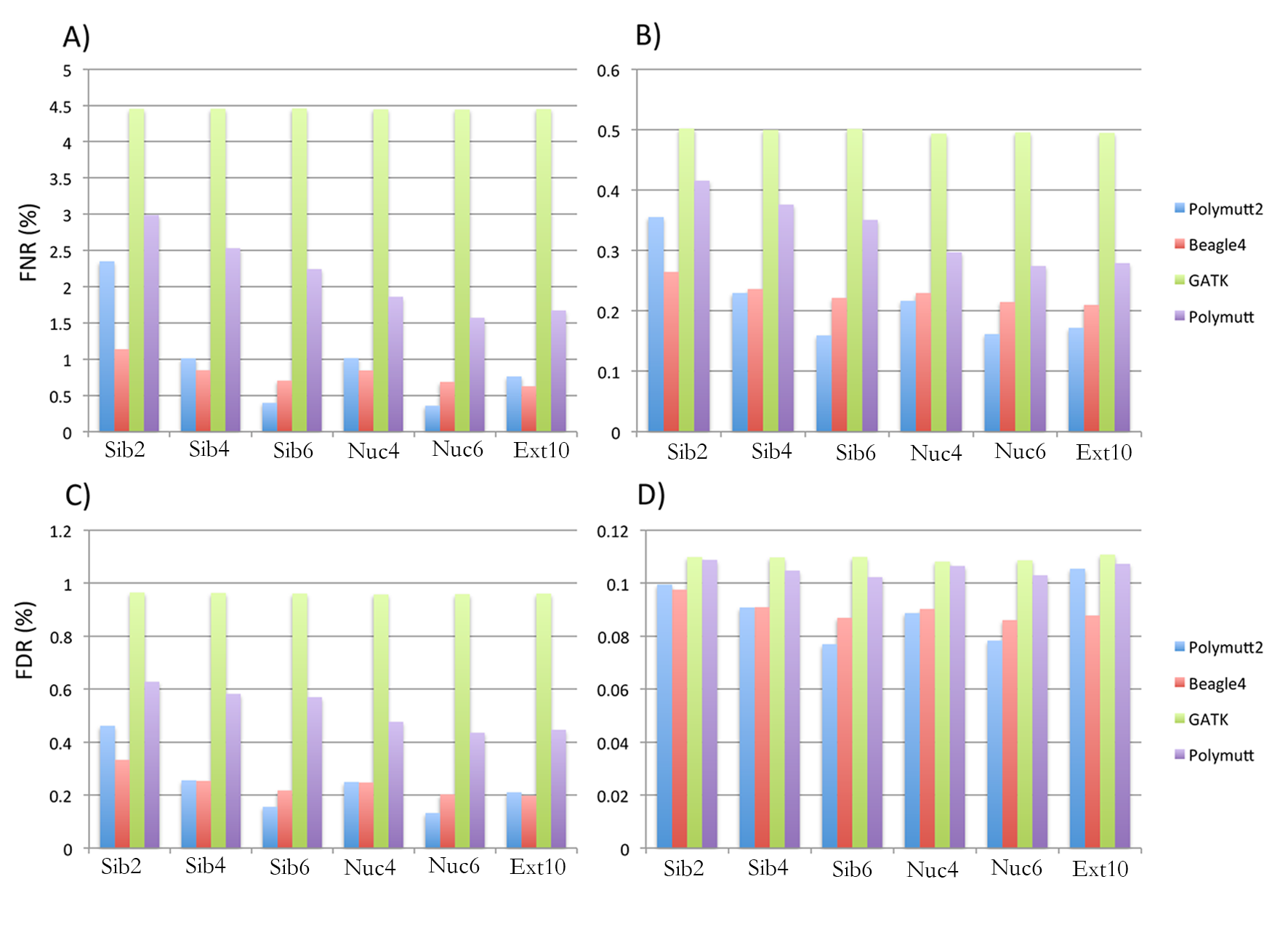

Supplement: S1 Fig — Panels A and B show FNR (%) for sequencing coverage of 10X and 20X, and panels C and D show the FDR (%) for the same set of coverage. (TIF) [file pgen.1005271.s001.tif]

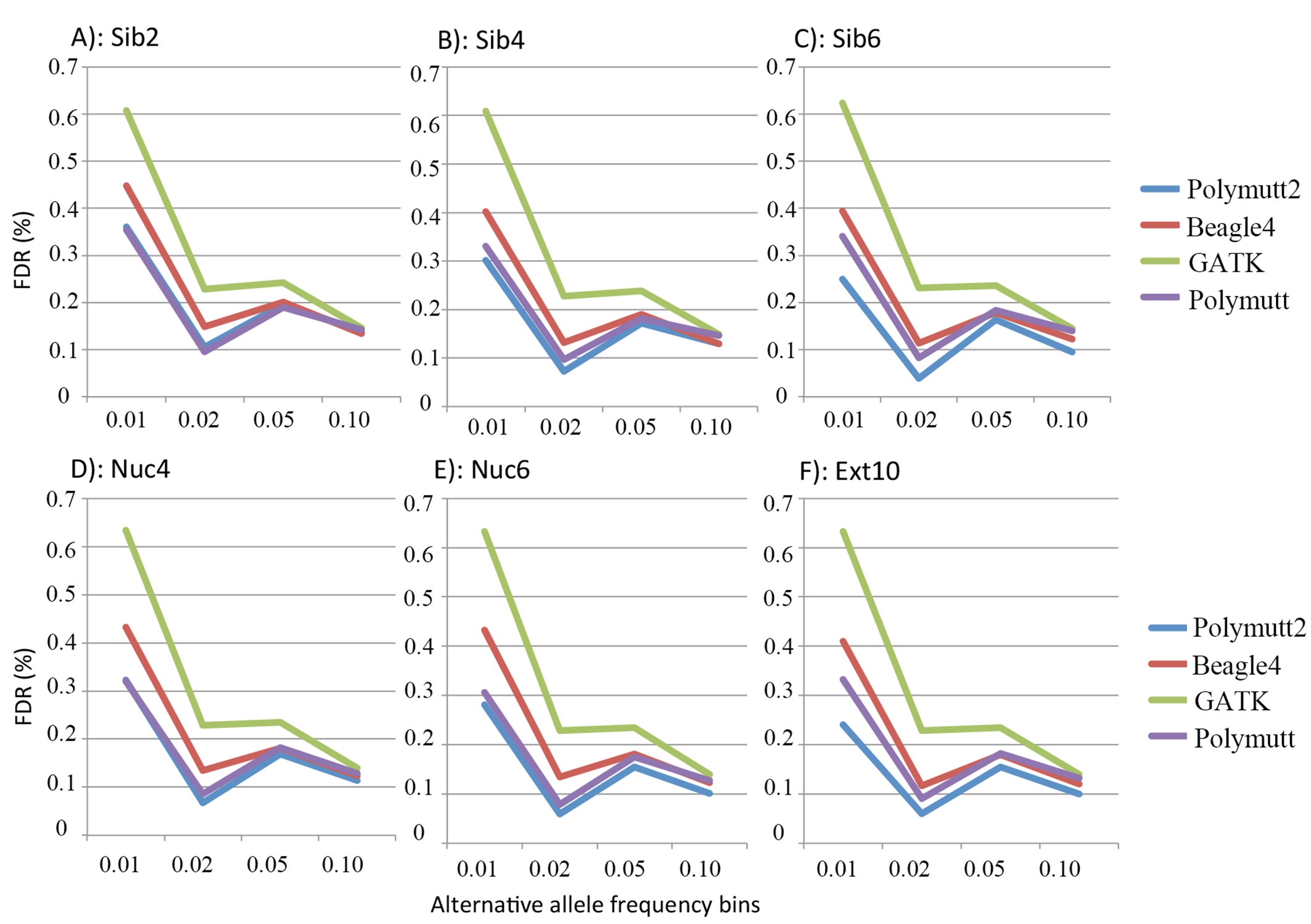

Supplement: S2 Fig — Results of different pedigrees are shown in panel A) for Sib2, B) for Sib4, C) for Sib6, D) for Nuc4, E) for Nuc6 and F) for Ext10. (TIF) [file pgen.1005271.s002.tif]

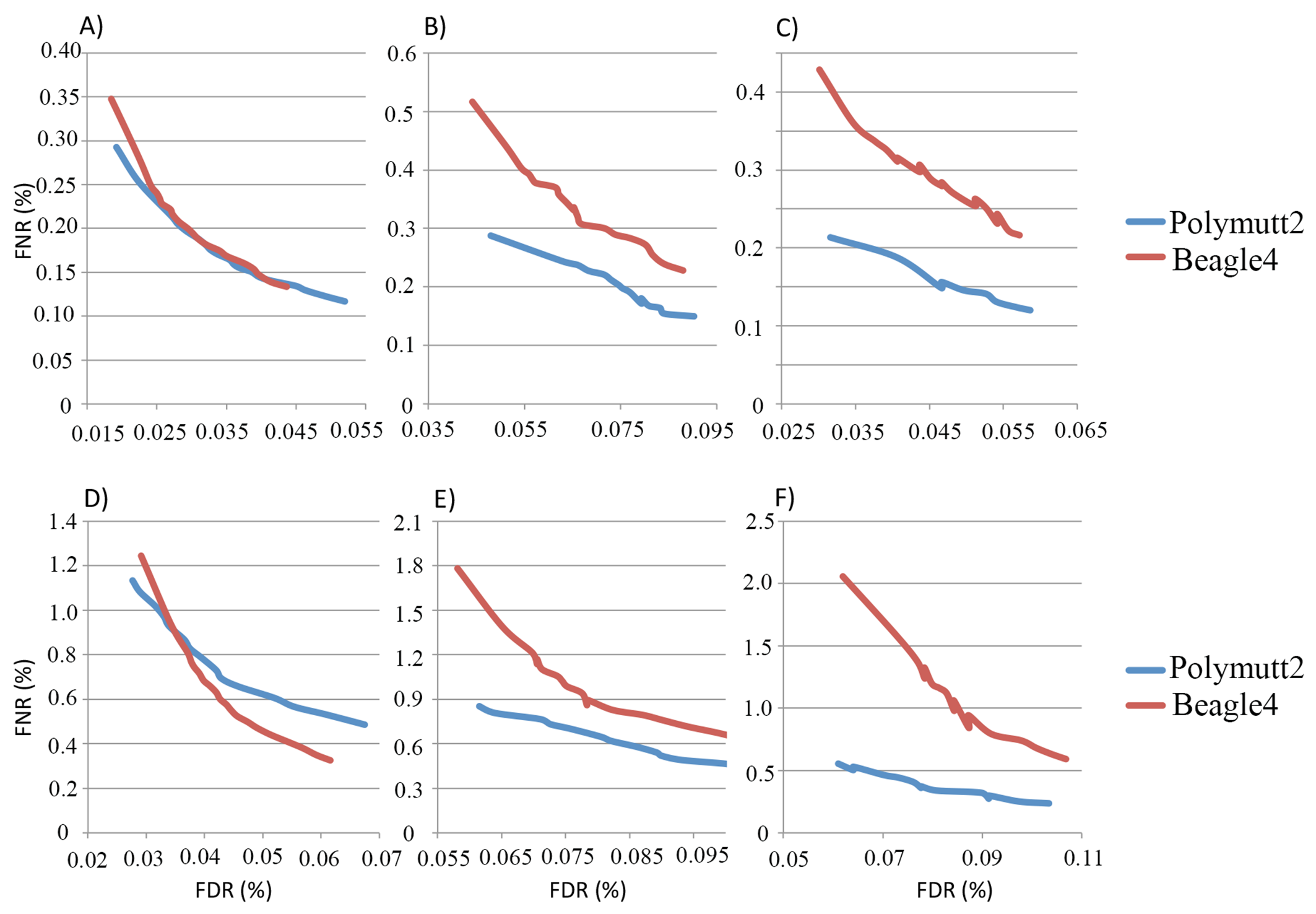

Supplement: S3 Fig — See Application to real data for the details on the pedigree and the calculation of error rates. (TIF) [file pgen.1005271.s003.tif]
